# Analysis of mutations in primary and metastatic synovial sarcoma

**DOI:** 10.18632/oncotarget.26416

**Published:** 2018-12-07

**Authors:** Zhuo Xing, Lei Wei, Xiaoling Jiang, Jeffrey Conroy, Sean Glenn, Wiam Bshara, Tao Yu, Annie Pao, Shinya Tanaka, Akira Kawai, Christopher Choi, Jianmin Wang, Song Liu, Carl Morrison, Y. Eugene Yu

**Affiliations:** ^1^ The Children's Guild Foundation Down Syndrome Research Program, Genetics and Genomics Program, Department of Cancer Genetics and Genomics, Roswell Park Cancer Institute, Buffalo, NY, USA; ^2^ Department of Biostatistics and Bioinformatics, Roswell Park Comprehensive Cancer Center, Buffalo, NY, USA; ^3^ Center for Personalized Medicine, Roswell Park Comprehensive Cancer Center, Buffalo, NY, USA; ^4^ OmniSeq Inc., Buffalo, NY, USA; ^5^ Department of Pathology, Roswell Park Comprehensive Cancer Center, Buffalo, NY, USA; ^6^ Department of Medical Genetics, Tongji Medical College, Huazhong University of Science and Technology, Wuhan, Hubei, China; ^7^ Department of Cancer Pathology, Hokkaido University Graduate School of Medicine, Sapporo, Japan; ^8^ Department of Musculoskeletal Oncology, National Cancer Center Hospital, Tokyo, Japan; ^9^ Center for Immunotherapy, Roswell Park Comprehensive Cancer Center, Buffalo, NY, USA; ^10^ Genetics, Genomics and Bioinformatics Program, State University of New York at Buffalo, Buffalo, NY, USA

**Keywords:** synovial sarcoma, SS18-SSX, metastasis, whole exome sequencing, ADAM 17

## Abstract

Synovial sarcoma is the most common pediatric non-rhabdomyosarcoma soft tissue sarcoma and accounts for about 8–10% of all soft tissue sarcoma in childhood and adolescence. The presence of a chromosomal translocation-associated *SS18-SSX*-fusion gene is causally linked to development of primary synovial sarcoma. Metastases occur in approximately 50–70% of synovial sarcoma cases with yet unknown mechanisms, which led to about 70–80% mortality rate in five years. To explore the possibilities to investigate metastatic mechanisms of synovial sarcoma, we carried out the first genome-wide search for potential genetic biomarkers and drivers associated with metastasis by comparative mutational profiling of 18 synovial sarcoma samples isolated from four patients carrying the primary tumors and another four patients carrying the metastatic tumors through whole exome sequencing. Selected from the candidates yielded from this effort, we examined the effect of the multiple missense mutations of ADAM17, which were identified solely in metastatic synovial sarcoma. The mutant alleles as well as the wild-type control were expressed in the mammalian cells harboring the *SS18*-*SSX1* fusion gene. The ADAM17-P729H mutation was shown to enhance cell migration, a phenotype associated with metastasis. Therefore, like ADAM17-P729H, other mutations we identified solely in metastatic synovial sarcoma may also have the potential to serve as an entry point for unraveling the metastatic mechanisms of synovial sarcoma.

## INTRODUCTION

Synovial sarcoma (SS) is a highly aggressive and distinct soft tissue sarcoma and the most common non-rhabdomyosarcoma soft tissue sarcoma in childhood and adolescence [1, 2]. Thirty percent of SS occur in patients younger than 20 years of age [3], and accounts for about 8–10% of all pediatric soft tissue sarcoma [4, 5]. Histological subtypes of SS include biphasic tumors containing epithelia-like/spindle cells, monophasic tumors containing only spindle cells, and poorly differentiated tumors [6]. At the genetic level, primary SS is associated with translocations between human chromosome 18 and X, t(X;18) (p11.2;q11.2), which may result in fusions between exon 10 of *SS18* and exon 6 of *SSX*. The consequence is the last 8 amino acid residues of SS18 are replaced by 78 amino acids from the C-terminal part of SSX, which could be SS18-SSX1, SS18-SSX2, or SS18-SSX4 [1, 2, 6–8]. 90–95% of SS carry chromosomal translocation-associated *SS18-SSX1* or *SS18-SSX2* fusion gene [1].

Treatment of SS includes surgical resection with or without radiotherapy for primary disease. SS is associated with local recurrence and distant metastases which occurs in approximately 50–70% of cases with yet unknown mechanisms [9]. Ifosfamide with or without doxorubicin has been used as chemotherapeutic agents for systemic treatment. Efficacy of chemotherapy for long-term survival remains uncertain, particularly for metastatic SS. The current five-year overall survival after metastasis is about 20–30% and few patients survive beyond 3 years in cases with multiple foci of metastatic disease [6]. In short, the prognosis of metastatic SS remains dismal and the vast majority of mortality is the result of metastatic disease and not the local recurrence. Therefore, there is an urgent need to identify genetic causes driving metastasis of this tumor type.

The increasing availability of massive parallel sequencing technology, including whole exome sequencing (WES), has led to generation of mutational profiles of primary and metastatic tumors affecting specific tissue/organ types. Such efforts have identified differential mutational profiles between primary and metastatic tumors for lung cancer [10], breast cancer [11], hepatocellular carcinoma [12], colorectal cancer [13, 14], and non-melanoma skin cancer [15], laying the ground for uncovering potential biomarkers and drivers for tumor progression and metastasis. In our present study, we extended this effort to SS for the first time and revealed the novel mutations on the affected genes detected solely in metastatic SS. To begin to analyze the impact of these mutations, we carried out the experiments to examine the effect of the metastatic SS-associated ADAM17 mutations on metastasis-related phenotype.

## RESULTS

### Identification of mutations in primary and metastatic synovial sarcoma by whole exome sequencing

**Figure 1:**
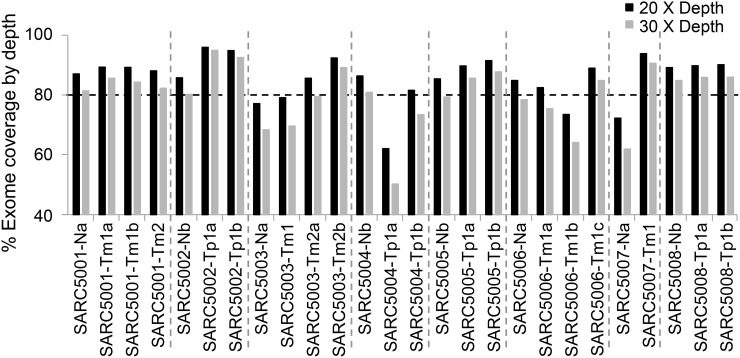
Whole exome sequencing coverage at 20X (black bar) and 30X (gray bar) depth Each bar on the x-axis represents a single sample and the percentage on the y-axis indicates the percentage of bases, out of all sequenced bases, that had at least 20X (black bar) or 30X (gray bar) coverage. The horizontal dash line marks the 80 percentage. Tp, Primary synovial sarcoma; Tm, Metastatic synovial sarcoma; N, Non-neoplastic tissue samples or blood samples from the same patients.

To identify mutations in tumors, we analyzed primary and metastatic SS isolated from patients along with matched normal tissues or blood samples using whole exome sequencing. Patients were divided into two groups represented by four patients who had primary SS and an additional four patients with metastatic disease. Follow-up for the patients with primary SS showed no evidence of metastatic disease after five years. As a note of reference, 80% of metastatic events often occur within the first two years after initial diagnosis for SS [16]. In the patient group representing non-metastatic SS, multiple segments of tissue were procured and sequenced. For patients in the metastatic group at least two separate foci of metastatic disease were procured and sequenced except for one patient. For one patient, this represented three separate foci of metastatic disease from the same surgical event, while for two other patients the separate foci of metastatic tumor were procured from two different surgical events. This approach of studying multiple segments of the same tumor or multiple tumors from the same patient facilitates identification of driver mutations. In total, whole exome sequencing was completed for 18 tumor samples and 8 normal tissue samples from 8 patients with SS (Figure 1). Most samples (21 out of 26) have at least 80% of the targeted regions covered by more than 20× coverage. Among the genes with the mutations identified (Supplementary Table 1), they include Cancer Gene Census genes and other cancer-associated genes with three of which carried multiple mutations (*ARID1B*, *CSMD1* and *ADAM17*) (Table 1) [17–25]. Importantly, a significant number of the mutations were detected solely in the metastatic SS (Table 1 and Supplementary Table 1), which include the missense mutations in ADAM17, ADAM17-P729H (nt: C2186A) and ADAM17-K805T (nt: A2414C) detected in Samples SARC5001 and SARC5003, respectively (Table 1 and Supplementary Table 1). Our data showed these ADAM17 mutations were present in six separate foci of metastatic tumors in two patients with each mutation from three foci in each patient (Figure 1; Supplementary Table 1).

**Table 1:** Examples of the mutations in cancer-associated genes detected in synovial sarcoma

Group	Primary SS				Metastatic SS				References
Sample	SARC5002	SARC5004	SARC5005	SARC5008	SARC5001	SARC5003	SARC5006	SARC5007	
**Number of mutations**	16	10	17	7	29	33	12	2	
***ADAM17***					a	a			Mochizuki and Okada 2007
***ARID1B***	a	a					c		Sausen *et al.* 2013; Wu *et al.* 2016
***CSMD1***		a	a		a				Ma *et al.* 2009; Shull *et al.* 2013
***ATM***					b				Futreal *et al.* 2004
***CRLF2***						a			Futreal *et al.* 2004
***CSNK2A1***				a					Bae *et al.* 2016
***DOT1L***				d					Kim *et al.* 2012; Wong *et al.* 2017
***JAK1***					a				Futreal *et al.* 2004
***KDM5C***					d				Futreal *et al.* 2004

a, missense mutation; b, nonsense mutation; c, deletion; d, frameshift mutation.

### Construction of the vectors expressing *ADAM17* and metastatic synovial sarcoma-associated *ADAM17* mutants

**Figure 2:**
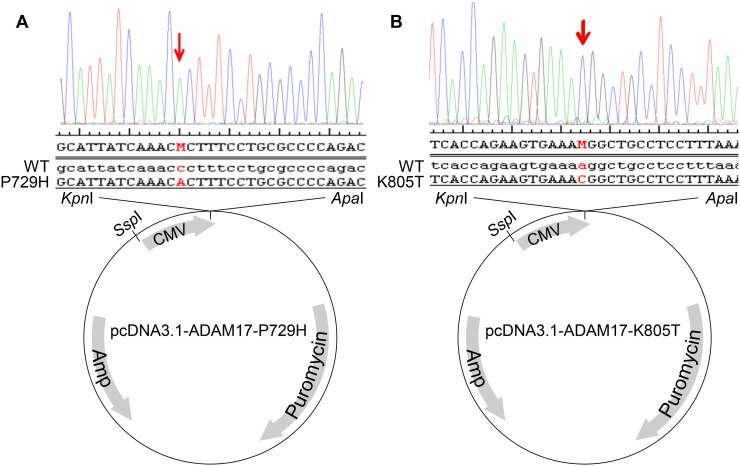
Construction of the expression vectors pcDNA3.1-ADAM17-P729H (**A**) and pcDNA3.1-ADAM17-K805T (**B**). Human ADAM17 full-length cDNA was amplified from the vector pRK5F-TACE using a high-fidelity DNA polymerase, which was then cloned into the Invitrogen TOPO vector. GeneArt Site-Directed Mutagenesis System (Invitrogen) was used to generate ADAM17-P729H and ADAM17-K805T mutations. The expression vectors pcDNA3.1-ADAM17-P729H and pcDNA3.1-ADAM17-K805T, along with the pcDNA3.1-ADAM17-WT were constructed by inserting the mutated ADAM17 and the wild-type ADAM17 full-length cDNA individually into KpnI and ApaI sites of pcDNA3.1-Puro+ expression vector. ADAM17 mutations in the vectors were confirmed using the following sequencing primers: CGC AAA TGG GCG GTA GGC GTG and TAG AAG GCA CAG TCG AGG.

Because ADAM17 has been extensively implicated in metastasis [18, 26–33] and its mutations detected in our multiple metastatic SS samples in different patients, but not in primary SS (Table 1), we decided to choose ADAM17-P729H and ADAM17-K805T in our first attempt to reveal the potential impacts of the mutations identified solely in metastatic SS. As a critical step of this effort, we constructed the expression vectors carrying *ADAM17* and the mutations identified. Human ADAM17 full-length cDNA was amplified from the vector pRK5F-TACE (Addgene) using Phusion High-Fidelity DNA Polymerase (NEB) [34], which was then cloned into the Invitrogen TOPO vector. ADAM17-P729H and ADAM17-K805T mutations were introduced through GeneArt Site-Directed Mutagenesis System (Invitrogen). The mutated ADAM17 and the wild-type ADAM17 full-length cDNA were then individually inserted into KpnI and ApaI sites of pcDNA3.1-Puro+ expression vector to generate the expression vectors pcDNA3.1-ADAM17-P729H and pCDNA3.1-ADAM17-K805T (Figure 2), in addition to the control vector pcDNA3.1-ADAM17-WT. Introduced mutations in the expression vectors were confirmed by sequencing using Primers CMV forward: CGC AAA TGG GCG GTA GGC GTG and BGH reverse: TAG AAG GCA CAG TCG AGG (Figure 2A and 2B). Before transfections, the expression vectors were linearized by cutting at SspI which is located proximal to the CMV promoter (Figure 2).

### Analysis of the impact of metastatic synovial sarcoma-associated *ADAM17* mutants on cell migration

**Figure 3:**
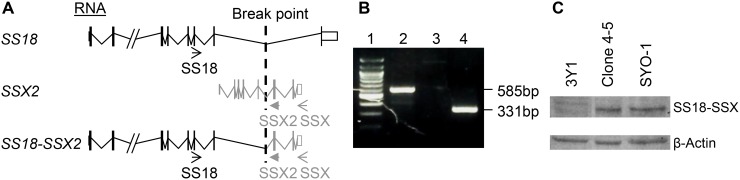
Expression analysis of the SS18-SSX fusions in cells. (**A**–**B**) The presence of the SS18-SSX2 fusion RNA was confirmed in SYO-1 cells. (**A**) The predicted RNA of SS18, SSX2 & SS18-SSX2 in human cells. Arrow heads: PCR primers. SS18: 5′-CAACAGCAAGATGCATACCA-3′, SSX: 5′-CACTTGCTATGCACCTGATG-3′, and SSX2: 5′-GGCACAGCTCTTTCCCATCA-3′. Break point of the genes in SYO-1 cells: a vertical dotted line. (**B**) RT-PCR results from cDNA of SYO-1 cells. Lane 1: DNA size markers; Lanes 2-4: Based on the primer pairs for SS18-SSX fusion (585bp), for SS18-SSX1 fusion (no band), for SS18-SSX2 fusion (331bp). (**C**) Western blot analysis of the SS18-SSX proteins. Lanes 1-3: Parental 3Y1 cells, Clone 4–5 expressing SS18-SSX1, SYO-1 cells expressing SS18-SSX2 using the antibody specific for human SSX (FL-188, sc-28697, Santa Cruz Biotechnology). β-Actin was used as a loading control, detected by β-Actin-specific antibody (AC-15, A1978, Sigma).

**Figure 4:**
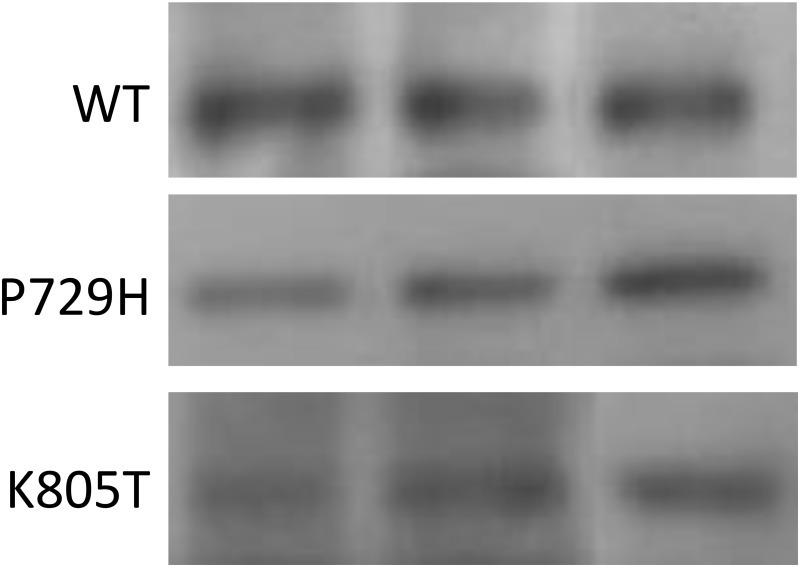
Western blot analysis Western blot analysis of the wild-type ADAM17 (WT), ADAM17-P729H, and ADAM17-K805T in three 3Y1 rat embryonic fibroblast cell clones which expressed both the fusion protein SS18-SSX1 and one of ADAM17 alleles, WT, P729H, or K805T. A mouse monoclonal antibody specific for human ADAM17 (B-6, sc-390859, Santa Cruz Biotechnology) was used, which did not detect the endogenous rat ADAM17 protein expressed in 3Y1 cells (see Supplementary Figure 3). Different lanes represent the samples isolated from different individual clones carrying one of three ADAM17 alleles.

It has been established that the presence of either translocation-associated SS18-SSX1- or SS18-SSX2 is causally linked to development of primary synovial sarcoma [1, 2, 6–8]. In addition, it has been shown that the expression of either SS18-SSX1 or SS18-SSX2 is sufficient to result in formation of synovial sarcoma in mouse models [35, 36]. These conclusions facilitate the experimental designs for analyzing potential metastatic drivers, such as ADAM17-P729H and ADAM17-K805T, from two different angles. First, the impact of a potential driver on metastasis will need to be analyzed in a model system carrying a SS18-SSX fusion oncogene. Second, a model system expressing either SS18-SSX1 or SS18-SSX2 is suited for analyzing the impact of a potential driver. Although a cell line derived from SS carry a SS18-SSX fusion oncogene, it often harbors additional genetic alterations. SYO-1 cell line is such an example. As one of the most widely used SS cell lines, SYO-1 also harbors a significant number of other genetic alterations, which include loss of a sex chromosome, del(3)(p13q21), t(6;6) (p23;q21), trisomy 7, trisomy 8, t(10;20)(q22;q11.2), del(11)(q23), and monosomy 13 [37]. Therefore, while we used SYO-1 as a control in this study, we did not use it for examining the impact of the ADAM17 mutations we detected on metastasis in order to avoid potential complications associated with interpretations of the results. Instead, we employed another cellular system, which has been successfully used for modeling SS by expressing a SS18-SSX fusion oncogene in 3Y1 rat embryonic fibroblast cells [38]. Here we adapted this system for analyzing the impact of the metastatic SS-associated mutations. We first transfected 3Y1 cells with pCXN2-SYT-SSX1, a vector expressing a SS18-SSX1 fusion gene (Supplementary Figure 2) [38], and isolated G418-resistant clones. One of such clones, Clone 4–5, showed a comparable level of expression of SS18-SSX when compared to SYO-1 cells (Figure 3). SYO-1 is a tumor cell line established using SS isolated from a patient, which carries a *SS18-SSX2* fusion oncogene (Figure 3A and 3B) [37]. The 67-kD SS18-SSX1 and SS18-SSX2 proteins expressed in Clone 4–5 and SYO-1 cells showed indistinguishable electrophoretic mobilities (Figure 3C), similar to SS18-SSX1 and SS18-SSX2 proteins expressed in COS-1 cells which is significantly larger than 30-kD SSX proteins [38, 39]. Afterwards, pcDNA3.1-ADAM17-WT, pCDNA3.1- pcDNA3.1-ADAM17-P729H or ADAM17-K805T was linearized with SspI and they were then transfected to Clone 4–5 cells individually. After the selection with puromycin, the individual survived clones were isolated. Expression of ADAM17 or its mutants was analyzed by western blot and the clones with the similar expression level were used for the subsequent experiment (Figure 4).

**Figure 5:**
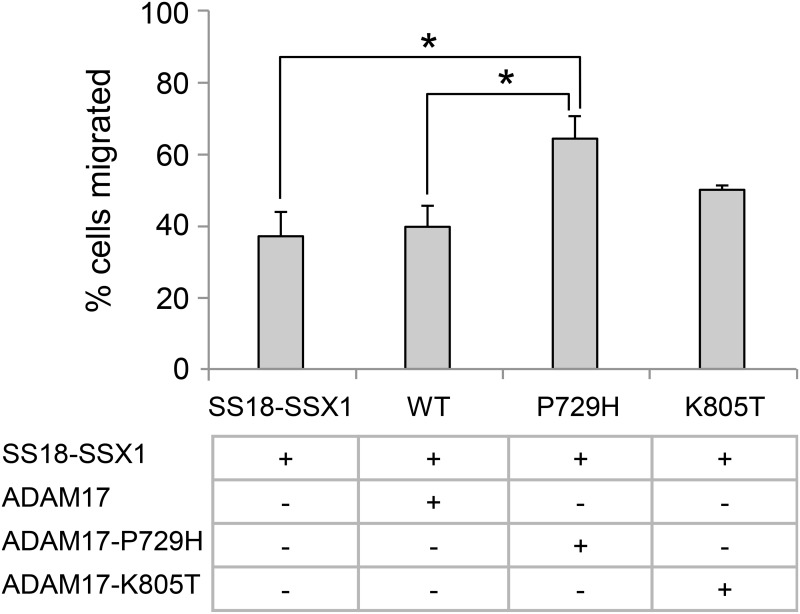
Analysis of the effects of metastatic synovial sarcoma-associated ADAM17 mutations using cell migration assay The graph represents the percentage of cells migrated after 17 h. All the cells carrying the *SS18-SSX1* fusion gene in addition to expressing the wild-type ADAM17, ADAM17-P729H, or ADAM17-K805T. All the results were are expressed as the means ± standard errors from three independent experiments. ^*^P < 0.05.

Accelerated cell migration is considered a key phenotypic feature associated with metastasis [40]. Therefore, we compared the migration rates among the pooled clones expressing the wild-type ADAM17, ADAM17-P729H or ADAM17-K805T. The result showed the clones expressing ADAM17-P729H or ADAM17-K805T have faster migration rates than the pooled clones expressing the wild-type ADAM17 (Figure 5). However, the difference is statistically significant only in the comparison between the pooled clones expressing ADAM17-P729H vs. the pooled clones expressing the wild-type ADAM17 (*P* = 0.037), but not statistically significant in the comparison between the pooled clones expressing ADAM17-K805T vs. the pooled clones expressing the wild-type ADAM17 (*P* = 0.170). These results suggest that ADAM17-P729H mutation may directly contribute to metastatic phenotype of SS while ADAM17-K805T may contribute to metastasis of SS through interaction with another factor(s).

## DISCUSSION

There were two previous studies of SS mutations at the genomic level, in which the Illumina human SNP microarray was used for mutation analysis of the primary SS [41] and the Ion AmpliSeq Comprehensive Cancer Panel was used for analysis of the sequences of pre-selected 409 genes in SS [42]. Different from these efforts, our study described here is the first comparative analysis of mutations associated with primary and metastatic SS using whole exome sequencing. That may partly explain why the genes with mutations we identified are different from those identified in the previous studies (Table 1 and Supplementary Table 1) [41, 42]. For example, no ADAM17 mutations were reported in either previous study, which may be because the primary SS samples were used in the study by Qi *et al.* and the Ion AmpliSeq Comprehensive Cancer Panel used by Vlenterie *et al.* does not include ADAM17 [41, 42]. For some members from a protein family with very similar structures which were implicated in these studies (Table 1 and Supplementary Table 1) [41, 42], such as CSMD proteins, our study revealed mutations could exist in both the primary and metastatic SS, suggesting the genes in these families are not exclusively mutated in the metastatic SS (Table 1 and Supplementary Table 1) [42]. Such information is important for prioritizing candidate genes for investigating their roles in metastasis.

It is interesting that the frequencies of mutations varied substantially among the SS samples from different patients. For example, the number of mutations is significantly lower in the tumor sample isolated from Patient SARC5007 when compared with other SS samples, even though it is a metastatic tumor. There are several possibilities for such an observation. First, critical mutations may be present in noncoding regions which are undetectable by whole exome sequencing but may underlie metastasis. Second, the small number of the mutations detected by whole exome sequencing may be sufficient to drive metastasis. Therefore, whole genome sequencing might be useful for distinguishing these possibilities.

Among the genes mutated multiple times in our SS samples, *ADAM17* is the only one which occurred solely in multiple patients with metastatic SS but not in patients with primary disease (Table 1). The members of the ADAM family are key mediators of cell signaling events which determine cellular fate, proliferation, and growth [43, 44]. ADAM17 is expressed in most tissues [45], and its unusual importance is illustrated by the fact that quadruple-knockout mice that lack ADAM9, ADAM12, ADAM15 and ADAM17 resemble *Adam17*−/− mice [31, 46]. ADAM17 catalyzes many critical events associated with tumor invasion and metastasis, including shedding or release of some of the most important protein ligands from transmembrane proteins [31, 44], and activation of Notch receptor [31]. ADAM17's role in promoting metastasis has also been supported by other *in vitro* and *in vivo* studies [26–29]. While the extracellular domain of ADAM17 is involved in substrate recognition and cleavage, it is interesting that the mutations we detected in metastatic SS are located in the cytoplasmic domain of this protein because the cytoplasmic domain contains phosphorylation sites, including Thr735, Ser791 and Ser819, and interacts with intracellular signaling molecules [47]. Phosphorylation of Thr735 likely mediates ADAM17 trafficking and is required for activation of ADAM17-catalyzed shedding by MAP/ERK kinase [26, 27]. Missense mutations close to phosphorylation sites or critical motifs are more likely to affect phosphorylation or motif interactions through conformational changes [48–52]. Because of the positional proximity, P729H may have an impact on phosphorylation/dephosphorylation of neighboring Thr735 [26]. P729H may also have an impact on the interaction between the PAPQTPGR motif (amino acids 731–738) of ADAM17 and MAD2, a critical mediator of the genome instability [53]. Therefore, ADAM17-P729H may act as a critical driver in metastasis of SS in the presence of SS18-SSX through one or more above-mentioned mechanisms. Ser819 in ADAM17 is a target for growth-factor-induced phosphorylation via MAPK/ERK, whereas Ser791 undergoes dephosphorylation in response to growth factor stimulation [28]. Because of the positional proximity, we speculated that ADAM17-K805T may have the impact on metastasis-associated cell migration via affecting phosphorylation and dephosphorylation of neighboring Ser791 and Ser819, respectively [26]. However, despite showing a tendency, the increase in cell migration caused by ADAM17-K805T is not statistically significant, suggesting alteration of another factor(s) may be needed in interacting with this mutation to facilitate metastasis of SS.

In addition to ADAM17, mutations of ARID1B and CSMD1 were identified in the SS samples isolated from 3 out of 8 cancer patients in this study. Although the sample size of our study is limited, it is still a surprise that over 37% of our tumor samples harbor mutations of these two genes. This frequency is significantly higher than what have been reported in other tumor types. For example, only 5 out of 72 cases of pediatric neuroblastoma harbor mutations of ARID1B [54] and only 5 out of 26 cases of colorectal cancers harbor mutations of CSMD1 [55]. Although both ARID1B and CSMD1 have been implicated in tumorigenesis [19, 20, 23, 54–56], our current data alone is not sufficient to implicate a link between ARID1B or CSMD1 mutations and metastasis because the mutations were identified in both primary and metastatic SS. However, on the other hand, we cannot exclude their possible impacts at this point because the mutations of these genes identified in metastatic SS are different from the mutations identified in primary SS, and it is possible that the mutations identified in metastatic SS may contribute to metastatic process. Therefore, the strategy described here for studying ADAM17 mutations could be used to analyze the impacts of individual ARID1B and CSMD1 mutations identified in metastatic SS on metastasis-associated phenotypes.

Metastatic SS remains a major challenge, and thus identification of metastatic drivers of this tumor type and unraveling of the associated metastatic mechanisms remain an urgent task. Our analysis of ADAM17 mutations described in this study demonstrates the mutations we identified solely in metastatic SS through whole exome sequencing are desirable candidates for the studies with the goal to understand the metastatic process of SS, which in turn can lay the foundation for rational development of novel therapeutic strategies to improve the prognosis of this deadly sarcoma.

## MATERIALS AND METHODS

### Patient samples

All patients were consented for specimen collection under a protocol approved by the Institutional Review Board (IRB) at Roswell Park Comprehensive Cancer Center (RPCCC), which also gave the approval for this study. The final diagnoses for tumor specimens were either primary or metastatic SS, supported by the FISH confirmation of the presence of t(X;18)(p11.2;q11.2). For Sample SARC5001, the presence of the *SS18*-*SSX1* fusion gene was confirmed by RT-PCR (Supplementary Figure 1). Adjacent non-neoplastic tissue samples or blood samples were obtained along with a section of the neoplasm. These tissues were collected according to the approved IRB protocol by the NCI-supported RPCCC Pathology Network Shared Resource (PNSR).

### Whole exome sequencing

The biospecimens were processed under the auspices of the PNSR and the associated clinical data were collected by the RPCCC Data Bank and Biorepository (DBBR). Once frozen, the tissue was transferred into a cryovial. Genomic DNA was extracted from these samples using the Qiagen FlexiGene kit (Valencia, CA). Nucleotide concentration of DNA samples was determined by both NanoDrop and PicoGreen techniques. DNA samples were stored at –80° C until analysis.

Genomic library construction was carried out using the Nextera DNA library Preparation Kit (Illumina) according to the manufacturer's instructions. 1 μg of DNA from each sample was fragmented to a size range of 150–200 bp, followed by end repair and adapters ligation. The size-selected product was PCR amplified. Libraries were purified and validated for appropriate size on a 2100 Bioanalyzer. The purified DNA libraries were hybridized with probes to exonic regions for 18 h at 65° C. The captured regions were then bound to streptavidin magnetic beads and washed to remove any non-specific bound products. The eluted library underwent a second PCR amplification to add sample specific barcodes to facilitate multiplexing. Final libraries were purified, validated by a bioanalyzer, and quantitated using a KAPA qPCR library quantitation kit (Kapa Biosystems). Libraries were then gone through clustering and sequencing on a HiSeq2500 sequencer according to the manufacturer's recommended procedure.

### Exome sequencing read mapping and detection of mutations

After passing the Illumina Real Time Analysis (RTA) filter, the sequence reads were aligned to the Human Reference Genome (NCBI build 37) using the Burrows-Wheeler Aligner [57]. After removing PCR duplicates using Picard (http://picard.sourceforge.net/), putative point mutations and small insertions and deletions (Indels) were identified using Bambino [58]. The identified putative mutations were further filtered to remove potential false calls as previously described [59]. All remaining calls were reviewed manually using the Bambino viewer [58]. The mutations were annotated using ANNOVAR [60] and the NCBI RefSeq sequence database.

### Expression vectors and vector construction

*SS18-SSX1* expression Vector pCXN2-SYT-SSX1 was described previously and SS18-SSX1 protein expressed by this vector demonstrated transforming activity in the cells which formed tumors in nude mice [38]. To express ADAM17, ADAM17-P729H, and ADAM17-K805T, the expression vectors pcDNA3.1-ADAM17-WT, pcDNA3.1-ADAM17-P729H, and pcDNA3.1-ADAM17-K805T were constructed, respectively (Figure 2; see Results section).

### Cell lines

3Y1 rat embryonic fibroblast cell line was purchased from Japanese Cancer Research Resources Bank. Clone 4–5 was established by transfecting 3Y1 cells with pCXN2-SYT-SSX1, the expression vector for the fusion oncoprotein SS18-SSX1. The cell clones expressing human wild-type ADAM17, ADAM17-P729H or ADAM17-K805T were established by transfecting Clone 4–5 cells individually with the expression vectors pcDNA3.1-ADAM17-WT, pcDNA3.1-ADAM17-P729H or pcDNA3.1-ADAM17-K805T, respectively. As a widely used SS cell line, SYO-1 was established from a primary SS [37].

### Cell culture and transfection

All cells were cultured in Dulbecco's modified Eagle's (DMEM) medium with 10% fetal bovine serum (FBS). Linearized expression vectors were transfected into cultured cells using Lipofectamine 3000 Reagent (Invitrogen) according to the manufacturer's recommendation. After 24 hours, cells were seeded in DMEM supplemented with 10% FBS for 48 hours. Culture medium was then supplemented with an appropriate antibiotic to select for the drug-resistant cells.

### RT-PCR

RT-PCR was used to analyze expression of *SS18-SSX* in SYO-1 cells or a patient SS sample. Total RNAs, isolated from the cells or a tumor sample using TRIzol Reagent (Life Technologies), were used to generate cDNA by Superscript version III reverse transcriptase (Life Technologies), which were then used as the templates for PCR experiments. The PCR reactions were carried out with the following amplification conditions: an initial activation and denaturation at 95° C for 10 min, followed by 40 cycles of denaturation at 95° C for 15 s, primer annealing and extension at 60 ° C for 1 min.

### Western blotting

Whole cell lysates were prepared in RIPA buffer (50 mM Tris-HCl, pH 7.4, 150 mM NaCl, 1 mM EDTA, 1% Triton X-100, 0.1% SDS, 0.5% sodium deoxycholate) with freshly added inhibitors (1 mM phenylmethylsulfonyl fluoride, 10 mM NaF, 1 mM Na3VO4) and protease inhibitor mixture (Roche Applied Science). The protein concentration was determined by the Bradford protein assay (Bio-Rad). Proteins were separated on 7.5% SDS-polyacrylamide gels and transferred onto polyvinylidene difluoride membranes (PerkinElmer Life Sciences). The membranes were blocked with 5% nonfat milk in TBST (150 mM NaCl, 100 mM Tris-HCl, pH 7.4, 0.1% Tween 20). The following primary antibodies were used as indicated: anti-SSX rabbit polyclonal antibody FL-188 (sc-28697, Santa Cruz Biotech) for detecting human SS18-SSX1 and SS18-SSX2 proteins in Clone 4–5 and SYO-1 cells, respectively; anti-human ADAM17 mouse monoclonal antibody B6 (sc-390859, Santa Cruz Biotech) for detecting human ADAM17 and its mutants; anti-β-Actin mouse monoclonal antibody AC-15 (A1978, Sigma) and anti-GAPDH mouse monoclonal antibody 6-C5 (sc-32233, Santa Cruz Biotech) for detecting rat and human β-Actin and GAPDH, respectively, as the loading controls. After washing and incubating with horseradish peroxidase-labeled anti-rabbit or -mouse IgG secondary antibodies, the blots were washed, incubated with Lumi-Light chemiluminescence reagent (Roche Applied Science), and digitally imaged using a Chemi-Genius2 Bio-Imager.

### Cell migration assay

Transwell migration assays were performed as described previously [61, 62]. 5 × 10^4^ cells in DMEM with 0.5% FBS were plated onto Transwell filters (8 μm pore size; Corning, Corning, NY) in the top chamber insert in a 24 well plate. The lower chamber in the plate contained DMEM with 10% FBS. The cells were allowed to migrate for 17 hrs before they were stained and quantified.

### Statistical analysis

Statistical analysis was performed using Student *t* test. The level of statistical significance was set at *P* = 0.05. Data are presented as Mean ± SEM.

## SUPPLEMENTARY MATERIALS FIGURES AND TABLES




