# Supplementary material for: Analysis of mutations in primary and metastatic synovial sarcoma
Source: Oncotarget. 2018 Dec 7;9(96):36878–88. doi: 10.18632/oncotarget.26416 (PMC6305143; doi:10.18632/oncotarget.26416)
Supplement: Supplementary file 1 [file oncotarget-09-36878-s001.pdf]

Analysis of mutations in primary and metastatic synovial sarcoma

SUPPLEMENTARY MATERIALS

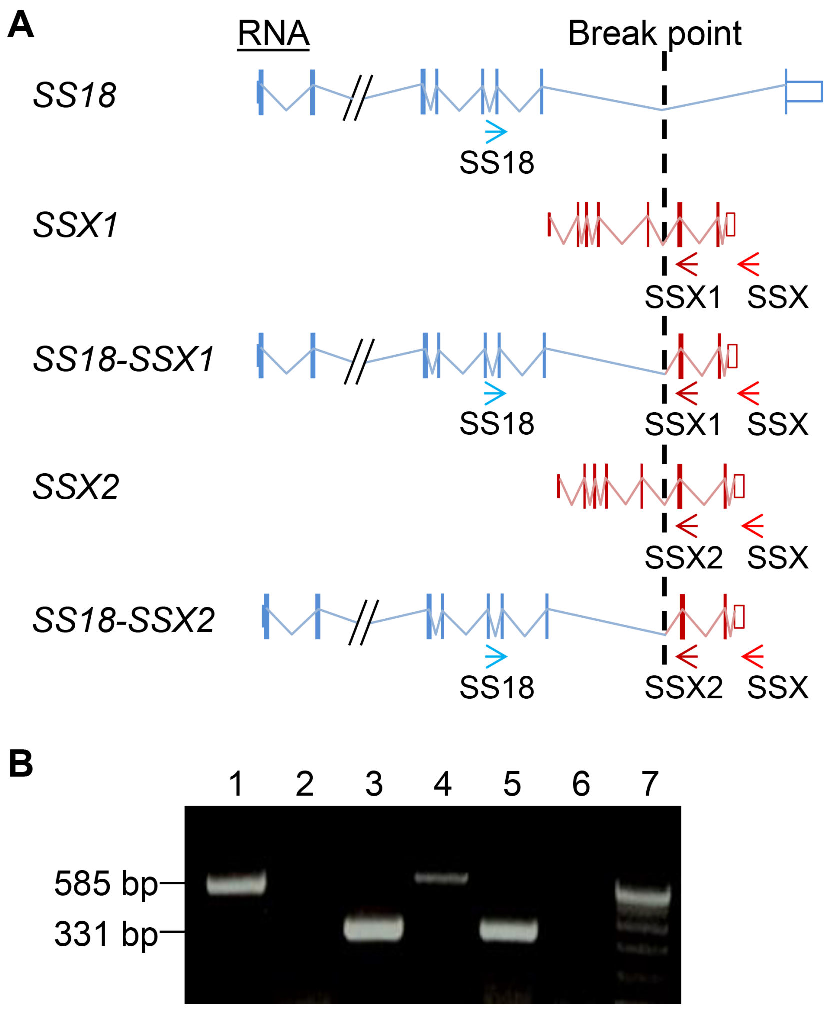

Supplementary Figure 1: Expression analysis of the *SS18-SSX* fusions in cells. (**A**–**B**) The presence of the SS18-SSX1 and SS18-SSX2 fusion RNA was confirmed in Sample SARC5001 and SYO-1 cells, respectively. (**A**) The predicted RNA of *SS18*, *SSX1*, *SS18-SSX1*, *SSX2*, and *SS18-SSX2* in human cells. Arrow heads: PCR primers. SS18: 5′- CAACAGCAAGATGCATACCA-3′, SSX: 5′-CACTTGCTATGCACCTGATG-3′, SSX1: 5′- GGTGCAGTTGTTTCCCATCG-3′, and SSX2: 5′-GGCACAGCTCTTTCCCATCA-3′. Break point of the genes in the tumor cells: a vertical dotted line. We confirmed SS18-SSX2 in SYO-1 cells and SS18-SSX1 in Sample SARC5001. (**B**) RT-PCR results from cDNA of SYO-1 cells (lane 1-3) and Sample SARC5001 (lane 4-6). Lanes 1 and 4: primer pair SS18-SSX (585bp); lanes 2 and 5: primer pair SS18-SSX1 (331bp); lanes 3 and 6: primer pair SS18-SSX2 (331bp); lane 7: DNA size markers.

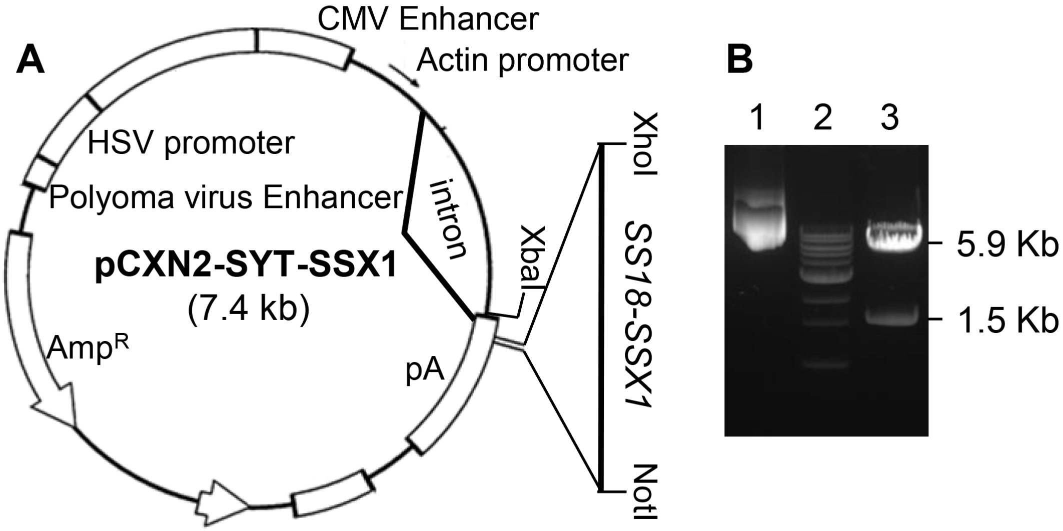

Supplementary Figure 2: The expression vector pCXN2-SYT-SSX1. (**A**) Plasmid map. (**B**) Digestion with XbaI and NotI shows a 1.5 kb band, which contains cDNA of the *SS18-SSX1* gene. Lanes 1–3: Undigested plasmid; DNA size markers; Digested with XbaI and NotI.

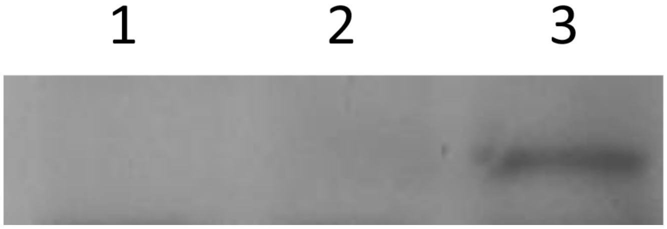

Supplementary Figure 3: Confirmation of the species specificity of antibody B-6 for detecting human ADAM17. Western blot analysis of the 3Y1 rat embryonic fibroblast cells (lane 1), Clone 4-5 cells expressing SS18-SSX1 (lane 2) and Clone 2 cells of Clone 4–5 expressing both SS18-SSX1 and ADAM17-P729H (lane 3) using mouse monoclonal antibody specific for human ADAM17 (B-6, sc-390859, Santa Cruz Biotech).

Supplementary Table 1: Mutations detected in the synovial sarcoma samples. See Supplementary_Table_1
