# Supplementary material for: Analysis of mutations in primary and metastatic synovial sarcoma
Source: Oncotarget. 2018 Dec 7;9(96):36878–88. doi: 10.18632/oncotarget.26416 (PMC6305143; doi:10.18632/oncotarget.26416)
Supplement: Supplementary file 2 [file oncotarget-09-36878-s002.pdf]

SUPPLEMENTARY MATERIALS

Supplementary Table 1: Mutations detected in the synovial sarcoma samples

SARC5001

Id(chr.posi.ref.mut)	Mut_ type	Gene	mRNA_acc	Class	AAChange	Effect	Cov_ Tm1a	Cov_ Tm1b	Cov_ Tm2	VAF_ Tm1a	VAF_ Tm1b	VAF_ Tm2	Confidence
1.65321345.G.T	SNV	*JAK1*	NM_002227	missense	Q499K	altering	28	25	17	0.39	0.32	0.00	high
1.200817824.G.T	SNV	*CAMSAP1L1*	NM_203459	missense	A643S	altering	57	27	29	0.00	0.00	0.10	low
2.9630595.G.T	SNV	*ADAM17*	NM_003183	missense	P729H	altering	47	49	44	0.40	0.47	0.43	high
2.98169667.C.-	Indel	*ANKRD36B*	NM_025190	frameshift	A413fs	truncating	199	62	79	0.40	0.39	0.52	high
3.32750166.A.G	SNV	*CNOT10*	NM_015442	missense	H95R	altering	51	28	32	0.53	0.57	0.41	high
3.36779606.C.T	SNV	*DCLK3*	NM_033403	missense	S182N	altering	197	237	216	0.37	0.47	0.48	high
3.50313745.G.A	SNV	*SEMA3B*	NM_004636	missense	E620K	altering	6	10	7	0.50	0.40	0.57	low
3.64004978.G.T	SNV	*PSMD6*	NM_014814	missense	A164D	altering	34	31	29	0.09	0.00	0.00	low
3.128182004.G.A	SNV	*DNAJB8*	NM_153330	missense	R29C	altering	84	112	143	0.46	0.50	0.52	high
3.169644462.G.T	SNV	*SAMD7*	NM_182610	missense	A138S	altering	122	139	142	0.41	0.44	0.51	high
3.174815020.C.A	SNV	*NAALADL2*	NM_207015	missense	P162T	altering	86	40	34	0.00	0.00	0.09	low
3.193385022.A.G	SNV	*OPA1*	NM_130837	missense	K979R	altering	211	202	180	0.42	0.40	0.42	high
4.123184696.G.A	SNV	*KIAA1109*	NM_015312	missense	R2361Q	altering	108	67	51	0.00	0.00	0.53	high
5.16781891.T.G	SNV	*MYO10*	NM_012334	missense	N217T	altering	247	222	180	0.40	0.40	0.38	high
5.16781892.T.A	SNV	*MYO10*	NM_012334	missense	N217Y	altering	242	212	173	0.39	0.38	0.36	high
6.135357859.C.T	SNV	*HBS1L*	NM_001145207	missense	R579H	altering	134	137	116	0.48	0.41	0.00	high
7.150499361.G.A	SNV	*TMEM176A*	NM_018487	missense	R78H	altering	128	143	137	0.40	0.43	0.50	high
8.3267055.T.A	SNV	*CSMD1*	NM_033225	missense	H545L	altering	52	22	29	0.50	0.41	0.00	high
10.104184466.T.C	SNV	*CUEDC2*	NM_024040	missense	D53G	altering	143	150	129	0.36	0.35	0.45	high
11.108202684.G.T	SNV	*ATM*	NM_000051	nonsense	E2570*	truncating	153	149	120	0.00	0.00	0.43	high
13.33111090.T.G	SNV	*N4BP2L2*	NM_014887	missense	K25N	altering	102	101	82	0.49	0.38	0.49	high
14.75375864.A.T	SNV	*RPS6KL1*	NM_031464	missense	W403R	altering	167	226	216	0.47	0.45	0.45	high
17.38176085.C.T	SNV	*MED24*	NM_014815	missense	E936K	altering	73	90	57	0.52	0.49	0.42	high
17.56565260.G.T	SNV	*HSF5*	NM_001080439	missense	H126N	altering	37	41	50	0.08	0.00	0.00	high
19.40366575.C.T	SNV	*FCGBP*	Unknown	splice	E30_splice	truncating	76	64	61	0.29	0.36	0.49	high

19.54314083.G.A	SNV	*NLRP12*	NM_144687	missense	A277V	altering	38	60	65	0.47	0.30	0.49	high
20.2729266.G.A	SNV	*EBF4*	NM_001110514	missense	R206H	altering	103	115	108	0.41	0.38	0.48	high
20.47768276.T.A	SNV	*STAU1*	NM_017453	missense	Y118F	altering	108	119	107	0.44	0.33	0.48	high
X.53230851.TA.-	Indel	*KDM5C*	NM_004187	frameshift	L647_I648fs	truncating	33	42	42	0.70	0.76	0.90	high

SARC5002

Id(chr.posi.ref.mut)	Mut_ type	Gene	mRNA_acc	Class	AAChange	Effect	Cov_ Tp1a	Cov_ Tp1b	VAF_ Tp1a	VAF_ Tp1b	Confidence
1.55224650.T.C	SNV	*PARS2*	NM_152268	missense	K62R	altering	152	100	0.42	0.42	high
1.228563774.C.T	SNV	*OBSCN*	NM_001098623	missense	R7618W	altering	412	231	0.24	0.24	high
3.42438795.C.T	SNV	*LYZL4*	NM_144634	missense	D135N	altering	85	63	0.46	0.40	high
3.123457770.G.A	SNV	*MYLK*	NM_053025	missense	P188S	altering	282	176	0.46	0.41	high
5.122729089.T.C	SNV	*CEP120*	NM_153223	missense	I239V	altering	208	166	0.46	0.45	high
5.141318200.C.T	SNV	*KIAA0141*	NM_014773	missense	T475I	altering	194	140	0.44	0.43	high
6.157521896.G.A	SNV	*ARID1B*	NM_020732	missense	D1390N	altering	184	111	0.41	0.43	high
9.86280060.T.C	SNV	*UBQLN1*	NM_013438	missense	M445V	altering	116	77	0.47	0.52	high
10.70266365.T.C	SNV	*SLC25A16*	NM_152707	missense	M112V	altering	166	129	0.48	0.53	high
11.4104687.A.T	SNV	*STIM1*	NM_003156	missense	D478V	altering	93	72	0.39	0.35	high
12.7301585.C.A	SNV	*CLSTN3*	NM_014718	missense	S622Y	altering	91	40	0.53	0.48	high
12.76424653.A.G	SNV	*PHLDA1*	NM_007350	missense	L290P	altering	241	115	0.41	0.39	high
16.2009743.A.T	SNV	*NDUFB10*	NM_004548	missense	T40S	altering	183	84	0.48	0.51	high
19.40902628.G.A	SNV	*PRX*	NM_181882	missense	P544L	altering	916	458	0.41	0.41	high
21.43510490.C.-	Indel	*UMODL1*	NM_001199527	frameshift	C219fs	truncating	329	183	0.42	0.45	high
X.86873057.T.A	SNV	*KLHL4*	NM_019117	missense	C284S	altering	129	86	0.19	0.16	high

SARC5003

Id(chr.posi.ref.mut)	Mut_ type	Gene	mRNA_acc	Class	AAChange	Effect	Cov_ Tm1	Cov_ Tm2a	Cov_ Tm2b	VAF_ Tm1	VAF_ Tm2a	VAF_ Tm2b	Confidence
1.17557146.T.G	SNV	*PADI1*	NM_013358	missense	L378R	altering	31	40	76	0.39	0.00	0.00	high
1.27874836.C.T	SNV	*AHDC1*	NM_00102988	missense	R1264Q	altering	37	35	61	0.00	0.37	0.54	high
1.70644587.G.A	SNV	*LRRC40*	NM_017768	missense	R251W	altering	38	109	160	0.32	0.41	0.46	high
1.152484108.C.A	SNV	*LCE5A*	NM_178438	missense	P33H	altering	34	27	85	0.00	0.11	0.00	low
1.197108981.G.C	SNV	*ASPM*	NM_018136	missense	P648A	altering	99	124	134	0.81	0.00	0.00	high
1.214171556.G.A	SNV	*PROX1*	NM_002763	missense	G560S	altering	17	15	41	0.00	0.67	0.63	high
1.248569858.G.A	SNV	*OR2T1*	NM_030904	missense	R188H	altering	42	118	170	0.00	0.41	0.34	high
2.9630367.T.G	SNV	*ADAM17*	NM_003183	missense	K805T	altering	71	106	190	0.46	0.51	0.41	high
2.48921430.G.T	SNV	*LHCGR*	NM_000233	missense	H294N	altering	55	156	198	0.51	0.43	0.45	high
3.10976836.C.A	SNV	*SLC6A11*	NM_014229	missense	P566Q	altering	25	22	50	0.12	0.00	0.02	low
3.46925118.C.A	SNV	*PTH1R*	NM_000316	nonsense	Y23*	truncating	14	11	34	0.14	0.09	0.00	low
3.134369750.A.G	SNV	*KY*	NM_178554	missense	V18A	altering	31	18	39	1.00	1.00	0.95	high
3.156422504.G.T	SNV	*TIPARP*	NM_00118471	missense	G520C	altering	13	35	61	0.15	0.00	0.02	low
5.145246191.A.T	SNV	*GRXCR2*	NM_00108051	missense	L146H	altering	165	239	370	0.00	0.43	0.49	high
6.10796304.T.-	Indel	*MAK*	NM_005906	frameshift	Q357fs	truncating	27	26	42	0.26	0.00	0.00	high
6.136582552.G.C	SNV	*BCLAF1*	NM_014739	missense	Q870E	altering	51	159	242	0.31	0.36	0.39	high
7.138968977.C.A	SNV	*UBN2*	NM_173569	missense	P1109Q	altering	12	44	87	0.17	0.00	0.01	low
9.100426706.C.A	SNV	*NCBP1*	NM_002486	missense	L628I	altering	80	159	259	0.15	0.00	0.00	high
9.135203548.C.G	SNV	*SETX*	NM_015046	missense	S1146T	altering	58	123	251	0.00	0.46	0.49	high
9.140173871.G.A	SNV	*C9orf167*	NM_017723	missense	A244T	altering	25	27	53	0.32	0.52	0.49	high
10.95399872.C.T	SNV	*PDE6C*	NM_006204	missense	R510C	altering	19	53	106	0.84	0.00	0.00	high
10.135139513.C.A	SNV	*CALY*	NM_015722	missense	G158C	altering	43	35	64	0.35	0.00	0.00	high
11.124744758.C.T	SNV	*ROBO3*	NM_022370	nonsense	Q676*	truncating	33	28	44	0.00	0.11	0.00	low
12.1943439.G.A	SNV	*LRTM2*	NM_00116392	missense	R222H	altering	21	25	37	0.00	0.52	0.59	high
12.40013068.C.A	SNV	*ABCD2*	NM_005164	missense	R117I	altering	131	216	313	0.00	0.44	0.46	high
12.53646957.T.A	SNV	*MFSD5*	NM_00117079	missense	L220Q	altering	173	187	350	0.40	0.00	0.00	high
12.101696321.AATTTAGTATT.-	Indel	*UTP20*	NM_014503	frameshift	N591_L594fs	truncating	75	155	255	0.00	0.03	0.11	high
12.129566381.C.G	SNV	*TMEM132D*	NM_133448	missense	V616L	altering	81	73	109	0.37	0.45	0.35	high
13.37678671.C.T	SNV	*CSNK1A1L*	NM_145203	missense	M241I	altering	114	221	411	0.35	0.00	0.00	high
15.25925416.A.T	SNV	*ATP10A*	NM_024490	missense	L1240M	altering	56	69	139	0.00	0.28	0.37	high

19.12187359.C.-	Indel	*ZNF844*	NM_00113650:	frameshift	P475fs	truncating	141	195	394	0.50	0.47	0.43	high
20.3641537.G.T	SNV	*GFRA4*	NM_145762	missense	P149Q	altering	32	23	80	0.09	0.00	0.01	low
X.1321372.G.A	SNV	*CRLF2*	NM_022148	missense	S128L	altering	262	238	380	0.50	0.49	0.44	high

SARC5004

Id(chr.posi.ref.mut)	Mut_ type	Gene	mRNA_acc	Class	AAChange	Effect	Cov_ Tp1a	Cov_ Tp1b	VAF_ Tp1a	VAF_ Tp1b	Confidence
4.88533645.G.T	SNV	*DSPP*	NM_014208	nonsense	E103*	truncating	15	71	0.13	0.00	Low
5.86564706.G.T	SNV	*RASA1*	NM_002890	missense	L146F	altering	8	6	0.13	0.33	Low
6.157405996.C.A	SNV	*ARID1B*	NM_020732	missense	S746R	altering	13	42	0.15	0.00	Low
7.130025014.C.A	SNV	*CPA1*	NM_001868	nonsense	S272*	truncating	17	25	0.00	0.12	Low
8.3263723.G.T	SNV	*CSMD1*	NM_033225	missense	H698N	altering	8	23	0.25	0.00	Low
9.136302998.G.A	SNV	*ADAMTS13*	NM_139025	missense	C522Y	altering	39	70	0.31	0.43	high
11.18382189.C.T	SNV	*GTF2H1*	NM_001142307	nonsense	R499*	truncating	62	169	0.32	0.41	high
12.112846242.G.C	SNV	*RPL6*	NM_001024662	nonsense	Y73*	truncating	77	177	0.08	0.00	Low
14.68046570.C.A	SNV	*PLEKHH1*	NM_020715	missense	H1054N	altering	31	70	0.10	0.00	Low
14.78189599.C.-	Indel	*SNW1*	NM_012245	frameshift	R352fs	truncating	120	191	0.47	0.44	high

SARC5005

Id(chr.posi.ref.mut)	Mut_ type	Gene	mRNA_acc	Class	AAChange	Effect	Cov_ Tp1a	Cov_ Tp1b	VAF_ Tp1a	VAF_ _Tp1b	Confidence
1.51253851.C.T	SNV	*FAF1*	NM_007051	missense	G63E	altering	101	75	0.55	0.51	high
1.62740108.C.-	Indel	*KANK4*	NM_181712	frameshift	R223fs	truncating	83	140	0.53	0.41	high
3.133305520.C.G	SNV	*CDV3*	NM_001134422	missense	Q194E	altering	77	66	0.51	0.53	high
4.74864293.G.T	SNV	*CXCL5*	NM_002994	missense	S2R	altering	169	355	0.02	0.15	high
5.133914626.T.-	Indel	*PHF15*	NM_015288	frameshift	C664fs	truncating	87	218	0.53	0.50	high
6.142691792.C.T	SNV	*GPR126*	NM_020455	nonsense	R311*	truncating	24	14	0.46	0.57	high
7.26225184.AGAG.-	Indel	*NFE2L3*	NM_004289	frameshift	R622_E623fs	truncating	98	62	0.37	0.27	high
7.6193548.A.G	SNV	*USP42*	NM_032172	missense	E788G	altering	24	67	0.42	0.39	high
7.6193549.A.C	SNV	*USP42*	NM_032172	missense	E788D	altering	24	62	0.42	0.37	high
8.3216814.C.T	SNV	*CSMD1*	NM_033225	missense	R1055Q	altering	163	268	0.61	0.53	high
8.74939043.A.G	SNV	*LY96*	NM_001195797	missense	I87M	altering	140	190	0.55	0.50	high
12.112667684.C.A	SNV	*C12orf51*	C12orf51	missense	V1941F	altering	17	10	0.06	0.20	low
14.34145420.G.A	SNV	*NPAS3*	NM_001164749	missense	E188K	altering	68	94	0.51	0.53	high
15.75942369.G.C	SNV	*SNX33*	NM_153271	missense	R309T	altering	71	154	0.48	0.49	high
19.3019415.G.C	SNV	*TLE2*	NM_003260	missense	T139S	altering	39	86	0.44	0.42	high
19.3913829.G.A	SNV	*ATCAY*	NM_033064	missense	V314I	altering	79	189	0.44	0.40	high
19.49621170.C.T	SNV	*LIN7B*	NM_022165	missense	S166L	altering	157	336	0.48	0.45	high

SARC5006

Id(chr.posi.ref.mut)	Mut_ type	Gene	mRNA_acc	Class	AAChange	Effect	Cov_ Tm1a	Cov_ Tm1b	Cov_ Tm1c	VAF_ Tm1a	VAF_ Tm1b	VAF_ Tm1c	Confidence
2.26414235.C.A	SNV	*HADHA*	NM_000182	missense	A726S	altering	45	27	92	0.00	0.11	0.00	low
2.43970979.G.T	SNV	*PLEKHH2*	NM_172069	missense	G1136C	altering	31	16	38	0.10	0.00	0.00	low
2.61298895.C.A	SNV	*KIAA1841*	NM_001129993	missense	T102N	altering	25	31	90	0.12	0.00	0.00	low
2.234652327.C.T	SNV	*DNAJB3*	NM_001001394	missense	G79D	altering	35	25	88	0.26	0.16	0.19	high
4.164393046.C.T	SNV	*TKTL2*	NM_032136	missense	R614K	altering	18	14	34	0.06	0.29	0.26	high
6.157099882.CGC.-	Indel	*ARID1B*	NM_020732	proteinDel	A273_A274>A	altering	38	25	43	0.00	0.16	0.02	high
8.17206580.G.A	SNV	*MTMR7*	NM_004686	missense	S160F	altering	31	18	68	0.26	0.22	0.18	high
11.639922.G.T	SNV	*DRD4*	NM_000797	missense	A225S	altering	25	26	47	0.00	0.12	0.00	low
11.63487442.G.T	SNV	*RTN3*	NM_201428	nonsense	E471*	truncating	37	9	53	0.00	0.22	0.02	low
14.104643584.G.T	SNV	*KIF26A*	NM_015656	missense	A1487S	altering	19	16	31	0.00	0.19	0.00	low
20.62839537.G.A	SNV	*MYT1*	NM_004535	missense	G330S	altering	31	14	30	0.32	0.21	0.33	high
22.27012243.G.A	SNV	*CRYBB1*	NM_001887	missense	A14V	altering	16	11	30	0.31	0.27	0.33	high

SARC5007

Id(chr.posi.ref.mut)	Mut_type	Gene	mRNA_acc	Class	AAChange	Effect	cov_Tm1	VAF_Tm1	Confidence
10.6533699.C.A	SNV	*PRKCQ*	NM_006257	nonsense	E246*	truncating	197	0.09	high
X.132091238.C.T	SNV	*HS6ST2*	NM_001077188	missense	R182H	altering	63	0.19	high

SARC5008

Id(chr.posi.ref.mut)	Mut_ type	Gene	mRNA_acc	Class	AAChange	Effect	Cov_ Tp1a	Cov_ Tp1b	VAF_ Tp1a	VAF_ Tp1b	Confidence
2.179647563.C.T	SNV	*TTN*	NM_133378	missense	V1024I	altering	62	59	0.39	0.10	high
3.38627355.C.-	Indel	*SCN5A*	NM_001099404	frameshift	D872fs	truncating	57	53	0.30	0.08	high
X.2853149.G.T	SNV	*ARSE*	NM_000047	nonsense	C498*	truncating	81	69	0.23	0.06	high
10.55849763.C.T	SNV	*PCDH15*	NM_001142771	missense	V665I	altering	137	129	0.31	0.18	high
19.2222119.C.-	Indel	*DOT1L*	NM_032482	frameshift	T984fs	truncating	218	179	0.30	0.13	high
19.14720877.TTCTGGGAGC.-	Indel	*CLEC17A*	NM_207390	frameshift	L282_A285fs	truncating	27	29	0.22	0.10	high
20.480571.T.G	SNV	*CSNK2A1*	NM_177559	missense	K74T	altering	199	194	0.29	0.14	high
